# The impact of adult children’s support on the psychological health of rural older adult people in China

**DOI:** 10.3389/fpubh.2023.1230580

**Published:** 2023-11-06

**Authors:** Juan Luo, Minglu Ji, Mengyuan Li, Anning Wang

**Affiliations:** School of Management, Shanghai University of Engineering Science, Shanghai, China

**Keywords:** children’s support, psychological health, rural older adults, children’s migration, China

## Abstract

**Background:**

Family old-age care is dominant in Chinese rural society, and children’s support is an important force in family old-age care. However, the migration of a large number of young and middle-aged rural laborers has undermined the traditional arrangements for old-age care in rural areas and affected the psychological health of the older adult.

**Methods:**

2014 China Longitudinal Aging Social Survey targets Chinese citizens aged 60 or older and covers 28 provinces in mainland China. In this paper, the database of the CLASS was selected for empirical analysis to explore the impact of children’s support on the depression level and loneliness of rural older adults through multiple linear regression, and was divided into two groups according to children’s migration to analyze heterogeneity.

**Results:**

Children’s financial support facilitates the maintenance of mental health among rural older adults. Children’s support promotes mental health among rural older adults, but this association does not exist among older adults without children’s migration. Individual characteristics of older people have a greater impact on mental health.

**Discussion:**

Our study firstly compares the differences of children’s migration status between children’s support and mental health among the older adult in rural China. In order to improve the mental health of the older adult, it is necessary to create a favorable atmosphere of love and respect for the older adult, improve the social security system in rural areas, and give full play to the strengths of the social forces, so as to ensure that the older adult have a sense of worthiness and enjoyment in their old age.

## 1. Introduction

In China, the degree of aging is much higher in rural areas than in urban areas. According to the Seventh National Population Census (Nov. 26, 2021), the proportion of older adult people over 60 years of age was 23.81% in rural areas while only 17.72% in urban areas.^1^ China has a tradition of filial piety for thousands of years, family old-age care has long been dominant in the urban and rural old-age care systems, especially in rural societies where the social old-age care system is not well developed (1), and children’s support plays an important role in family old-age care.

Dependence on family for old age is a Chinese tradition, children’s support plays an important role in the mental health of older people, but current research has come to different conclusions. One view is that children’s support is beneficial to the older adult in maintaining their mental health. Scholars have found that children’s financial support can increase the social participation of the older adult (2, 3), and that emotional interactions and caregiving can reduce the negative emotions of the older adult (4–7), thus enabling them to maintain their mental health. In addition, children’s support can increase the level of subjective well-being of the older adult (8), and enhance their spontaneity, willpower and integrity (9). Another viewpoint suggests that intergenerational supportive behaviors of children may have a negative impact on the mental health of older adults. Excessive support from children fails to maintain respect for older people (10), and violates their personal privacy (11). Daily caregiving by children may reduce the ability of older adults to live autonomously, ultimately decreasing their life satisfaction and increasing their depression (8, 12–14). Furthermore, older persons receive financial support from their children, which may violate their traditional role as breadwinner and perceive themselves as a burden to their children, leading to an increased sense of powerlessness and psychological burden on older persons (15, 16).

Since the reform and opening up of China, a large number of young and middle-aged rural workers have moved to the cities to work and do business, and the incomplete nature of large-scale urban–rural migration has prevented rural family members, especially parents, from moving with their adult children (17), increasing the physical distance between generations (18). The separation of parents’ and children’s living space makes the traditional style of family eldercare no longer realistic, destroying traditional Chinese family structures and endowment arrangements (1), making the older adult more prone to depression (19), and jeopardizing their physical health due to lack of care (20, 21). In rural China, the migration of children has made the burden of agricultural labor on the older adult heavy, widening the gap between the financial capital of the older adult and that of their children, and damaging the self-esteem of the older adult (22, 23). On the other hand, children’s migration also has favorable effects on parents. Migration allows older people to spend more time with friends and to participate actively in social activities (24). Adult children are able to provide other forms of support to their parents, for example, the economic and knowledge transfers of migrant children can expand household budgets (1), promote risk management strategies, and increase access to health care (25, 26). Migrating children significantly increase the willingness of parents left behind to participate in mutual support for the older adult and promote the development of new models of old-age care (27).

Throughout the existing literature, it can be seen that established studies generally recognize the role of children’s support on the mental health of the older adult, but related studies have not reached a consensus conclusion on the impact of children’s support on the mental health of the older adult. And the mass migration of children has evolved rapidly in response to social change, which has caused an impact on the function of family care for thousands of years in China, and it is especially true in rural areas, and the impact of children’s support on the mental health of rural older adults in this context, there is a paucity of relevant research, so this paper intends to use the data from the 2014 China Longitudinal Aging Social Survey to analyze the impact of children’s support on the mental health of rural older adults with different migration status of children.

## 2. Methods

### 2.1. Data sources

This paper uses data from the 2014 China Longitudinal Aging Social Survey (CLASS). The survey targets Chinese citizens aged 60 or older and covers 28 provinces (autonomous regions and municipalities directly under the central government) in mainland China. The questionnaires were collected through household interviews, and a total of 11,511 resident questionnaires were completed. The individual questionnaires for the older adult specifically collect information on the marital status, health status, retirement planning, economic status, family and children of the older adult aged 60 or older. This study mainly uses the information of older adult personal data in CLASS 2014, firstly screening the older adult population living in rural areas, and then screening the data according to the level of depression in the older adult, loneliness, children’s support, and other important research content of this paper, and finally get the effective sample data of 4,085.

In order to study the impact of intergenerational support on the psychological health of rural older adults in the context of children’s migration, we defined migrant children as children whose residence are not in the same township as that of his or her older adult parents, and categorized older adults into two main groups based on their status of having or not having migrant children, namely, have not migrant children and have migrant children, with sample data of 1,047 and 3,038, respectively.

### 2.2. Variable setting

#### 2.2.1. Dependent variable: mental health

The dependent variable studied in this paper is the mental health of older adults, which consists of two main dimensions: depression level and loneliness. The CES-D (Center for Epidemiological Survey-Depression Scale) is a common tool widely used around the world to screen for depressive symptoms in the general population, and has good reliability and validity (28, 29). Nine questions from the CES-D were included in the CLASS questionnaire, by which depression levels were measured, the responses were assigned a value, and the scores of each question were summed up, with higher scores representing a more severe tendency to depression in older adults. Russell developed the third edition of the UCLA(University of California at Los Angels) Loneliness Scale (UCLA-3) in 1996, and it was found that the UCLA-3 had good internal consistency, test-retest reliability and discriminant validity (30, 31). The CLASS questionnaire includes three UCLA-3 sub-questions, which were used to measure loneliness in this study, scored in the same way as the depression scale, and the scores for each question were summed, with higher scores representing higher levels of loneliness in older adults. According to the test, the Cronbach’s alpha of the depression scale and loneliness scale were 0.917 and 0.918, respectively, with good reliability (Table 1).

**Table 1:** Cronbach’s alpha of the depression scale and the loneliness scale.

	Cronbach’s alpha
Depression scale	0.917
Loneliness scale	0.918

#### 2.2.2. Independent variables: children’s support

Children’s support is usually analyzed from the perspective of social support, which can be divided into economic support, labor force support and emotional support, economic support is the monetary support given to the older adult by their children, labor force support is the life care of the older adult by their children, and emotional support is the emotional comfort of the older adult by their children (32). Therefore, this paper categorizes children’s support into three dimensions: financial support, life support, and emotional support.

In the CLASS database, ask for information on up to five children of older persons. The older adult received intergenerational support from multiple children, but the questionnaire does not cover the depressive level and loneliness of the older adult when receiving support from a specific child. These two variables represent the overall psychological well-being of the older adult, and this paper examines older adults’ holistic perceptions of intergenerational support from their children. If samples are paired with the older adult and a specific child, it would result in different children’s support all pointing to the same mental health status of older adults, which is inconsistent with existing research findings and departing from the integrity of older people’s cognition, so in this paper, children’s support is summed to measure the intensity of support for the older adult.

Financial support was examined and valued using the questionnaire question “In the past 12 months, has a child given you any money, food, or gifts, and what was the total value of these items?”, life support was measured and valued by the question “How often in the past 12 months has a child been able to help you with household chores?”, and the emotional support was measured and valued by the question “Do you feel that this child does not care enough about you?”. The questionnaire asked up to five children about their support for the older adult, and the support of each child was summed to get the scores for the intensity of the children’s financial support, life support, and emotional support, respectively.

#### 2.2.3. Control variables

Based on research needs, the control variables in this paper cover the main socio-demographic characteristics variables of the older adult, including age, gender, and marital status, the socio-economic status variables, including education level, and income-generating jobs, and the health status of the older adult, including self-assessed health status, chronic diseases, and the basic activity of daily living (BADL). The following table shows the scores of all variables (Table 2).

**Table 2:** Variable definitions and scores.

Variable settings	Score description
Dependent variable	
Depression level	Never = 0; sometimes = 1; often = 2 (continuous variable, 0–18)
Loneliness	Never = 0; sometimes = 1; often = 2 (continuous variable, 0–6)
Independent variables	
Children’s support	
Economic support	Not given = 0; 1–199 yuan = 1; 200–499 yuan = 2; 500–999 yuan = 3; 1,000–1999 yuan = 4; 2,000–3,999 yuan = 5; 4,000–6,999 yuan = 6; 7,000–11,999 yuan = 7; 12,000 yuan and above = 8 (continuous variable, 0–40)
Life support	Almost nothing = 0; several times a year = 1; at least once a month = 2; at least once a week = 3; almost every day = 4(continuous variable, 0–20)
Emotional support	Often = 0; Sometimes = 1; Occasionally = 2; Never = 3(continuous variable, 0–15)
Control variables	
Sociodemographic characteristics	
Gender	Female = 0; male = 1
Age	continuous variable, 57–98 years
Marital status	widowed/divorced/unmarried = 0; Married with spouse = 1
Socioeconomic status	
Educational attainment	primary school and below = 0; Junior high school and above = 1
Employment	None = 0; Yes = 1
Health status of the older adult	
Self-rated health	Unhealthy = 0; General =1; Health = 2
Chronic disease	None = 0; Yes = 1
BADL	Cannot move at all = 0; needs some help = 1; does not need help from others = 2 (continuous variable, 0–22)

### 2.3. Research methods

The study selected the cross-sectional data of CLASS database in 2014, and through the variance inflation factor test, the VIF values are all less than 5, which means that there is no serious multicollinearity relationship among the variables, and the regression analysis can be performed by multiple linear regression model. In this study, the empirical analysis was conducted through a linear regression model with multiple independent variables, with depression level and loneliness in old age as dependent variables, and children’s financial, life, and emotional support as independent variables. In the regression analysis, the multiple linear regression model was used to analyze the linear correlation between the dependent and independent variables, and the model is as follows:

$$Y=\beta_{0}+\beta_{1}X_{1}+\beta_{2}X_{2}+\ldots +\beta_{n}X_{n}+\varepsilon$$

In the above equation, Y is the dependent variable, X_1_, X_2_......X_n_ are the independent variables, ε is the random error term, β_0_ is the regression intercept, β_1_, β_2_......β_n_ are the regression coefficients.

This study first analyzes the differences in mental health between the full sample and the sub-sample of rural older adults with and without children’s migration in terms of depression level and loneliness using descriptive statistics to preliminarily determine the impact of child relocation on the mental health status of rural older adults. Then through the full sample multiple linear regression analysis of the mental health of rural older adult to explore the impact of children’s support on the psychological status of rural older adult, and finally through the sub-sample multiple linear regression analysis to further explore whether there is a difference in the impact of children’s support on the mental health status of rural older adult with or without children’s migration. This paper uses Stata16.0 software for regression analysis.

## 3. Results

### 3.1. Descriptive statistics

In terms of depression level and loneliness, the scores of the older adult without children’s migration are higher than those of the older adult with children’s migration. In terms of children’s support, the children’s financial support of the older persons without children’s migration is lower than that of the older persons with children’s migration, but children’s life support and emotional support of the older persons without children’s migration are higher than that of the older persons with children’s migration. In terms of personal characteristics, the marital status of the older adult with children’s migration is better than that of the older adult without children’s migration. In terms of socio-economic status, the average education level of older persons without children’s migration is lower, but more older persons without children’s migration still earn income through work. In terms of self-assessed health and chronic disease status, older persons with children’s migration are in poorer health, but have higher scores on BADL and are better able to take care of themselves (see Table 3).

**Table 3:** Descriptive statistics of variables.

Variable	Collectivity	Have children migrated (*N* = 3,038)	Haven’t children migrated (*N* = 1,047)
Dependent variables			
Depression level (mean)	9.80 (0.094)	9.69 (0.108)	10.13 (0.190)
Loneliness(mean)	2.15 (0.031)	2.11 (0.062)	2.25 (0.036)
Independent variables			
Children’s support			
Economic support (mean)	12.08 (0.077)	12.29 (0.090)	11.44 (0.144)
Life support (mean)	6.16 (0.055)	5.69 (0.058)	7.50 (0.120)
Emotional support (mean)	6.96 (0.045)	6.92 (0.052)	7.09 (0.090)
Control variables			
Sociodemographic characteristics			
Gender(%)			
0 = female	51.41%	50.69%	53.49%
1 = male	48.59%	49.31%	46.51%
Age(mean)	70.39 (0.125)	70.23 (0.143)	70.85 (0.257)
Marital status(%)			
0 = widowed/divorced/unmarried	37.31%	35.52%	42.5%
1 = Married with spouse	62.69%	64.48%	57.5%
Socioeconomic status			
Level of education(%)			
0 = primary school and below	86.71%	86.41%	87.58%
1 = Junior high school and above	13.29%	13.59%	12.42%
Employment(%)			
0 = none	67.05%	66.06%	69.91%
1 = yes	32.95%	33.94%	30.09%
Health status of the older adult			
Self-rated health(%)			
0 = Unhealthy	37.89%	38.45%	36.29%
1 = Average	25.04%	24.82%	25.69%
2 = Healthy	37.06%	36.73%	38.01%
Chronic disease(%)			
0 = none	21.27%	20.38%	23.88%
1 = yes	78.73%	79.62%	76.12%
BADL (mean)	11.16 (0.216)	11.18 (0.238)	11.11 (0.486)

### 3.2. Regression analysis

Specifically, from the regression results of the full sample of rural older adult, children’s financial support has a significant negative effect on the level of depression and loneliness of the older adult population (*p* < 0.01), with a greater effect on the level of depression. For every unit increase in children’s financial support, the level of depression and loneliness of the older adult population decreases by 0.106 units and 0.038 units respectively, namely, the higher the number of children’s financial support received by the older adult, the better the mental health of the older adult. In terms of children’s life support, children’s life support has a significant negative effect on loneliness in the older adult population (*p* < 0.05). For every unit increase in children’s life support, the loneliness of the older adult population were reduced by 0.017 units, namely, the higher the intensity of children’s life support received by the older adult, the more the loneliness of the older adult was weakened. And children’s emotional support had a negative effect on both depression levels and loneliness in older adults, but it is not statistically significant. In terms of control variables, male rural older adults have better mental health, rural older adults with higher education levels have better mental health, older adults with better self-assessed health and no chronic diseases have better mental health, and basic activity ability has a non-significant effect on the mental health of older adults (see Table 4).

**Table 4:** Full-sample multiple linear regression results of children’s support on the psychological welfare of rural older adult (*N* = 4,085).

Variable	Depression level	Loneliness
Economic support	−0.106*** (0.017)	−0.038*** (0.006)
Life support	−0.025 (0.024)	−0.017** (0.008)
Emotional support	0.047 (0.029)	0.014 (0.010)
Gender		
Female	Reference	
Male	−2.367*** (0.179)	−0.679*** (0.061)
Age	0.122*** (0.012)	0.037*** (0.004)
Marital status		
Widowed/divorced/unmarried	Reference	
Married with spouse	−0.990*** (0.198)	−0.601*** (0.067)
Level of education		
Primary school and below	Reference	
Junior high school and above	−2.104*** (0.256)	−0.602*** (0.087)
Employment		
None	Reference	
Yes	−0.101 (0.196)	0.044 (0.066)
Self-rated health		
Unhealthy	Reference	
Average	−1.614*** (0.218)	−0.427*** (0.074)
Healthy	−2.632*** (0.207)	−0.610*** (0.070)
Chronic disease		
None	Reference	
Yes	0.709*** (0.215)	0.188** (0.073)
BADL	0.021 (0.060)	0.005 (0.020)
Constant	4.969*** (1.220)	0.933** (0.414)
*R^2^*	0.222	0.193

*p < 0.1, **p < 0.05, ***p < 0.01.

### 3.3. Sub-sample regression analysis of children’s migration

As the level of social support and medical insurance for older people in rural China is relatively poor compared with that in urban areas, children are often the main providers of support for their old age, and large-scale urban–rural migration has resulted in an increase in the separation of adult children from their older adult parents, which may disrupt this old-age care arrangement and affect the psychological well-being of older people in rural areas. Therefore, this paper constructs a grouped comparative linear regression model of the impact of children’s support on the mental health status of the older adult population, to study in depth the factors related to the mental health status of the older adult population with migrant children.

Regarding the effect of children’s support on the mental health of rural older adults, the regression results showed that for older adults with children who migrated, financial support had a significant negative effect on the depression level of older adults (*p* < 0.01), while for older adults without migrated children, financial support did not have a significant effect on the depression level of rural older adults, and the effect on loneliness was also no more significant than for older adults with migrated children. Life support has a significant negative effect on loneliness only for the rural older adult with children migrated (*p* < 0.01). Emotional support from children has no significant effect on the depression level and loneliness of the older adult with or without children’s migration, which is consistent with the regression results of the full sample (see Table 5).

**Table 5:** Grouped multiple linear regression results of children’s support on the psychological welfare of rural older adult.

Variable	Have children migrated (*N* = 3,038)		Haven’t children migrated (*N* = 1,047)	
	Depression level	Loneliness	Depression level	Loneliness
Economic support	−0.117*** (0.020)	−0.042*** (0.007)	−0.069* (0.037)	−0.026** (0.012)
Life support	−0.049 (0.030)	−0.032*** (0.010)	−0.002 (0.044)	0.007 (0.015)
Emotional support	0.051 (0.033)	0.016 (0.011)	0.032 (0.058)	0.007 (0.019)
Gender				
Female	Reference			
Male	−2.419*** (0.206)	−0.670*** (0.070)	−2.210*** (0.360)	−0.695*** (0.120)
Age	0.126*** (0.014)	0.035*** (0.005)	0.112*** (0.024)	0.039*** (0.008)
Marital status				
Widowed/divorced/unmarried	Reference			
Married with spouse	−0.942*** (0.229)	−0.645*** (0.078)	−1.030*** (0.395)	−0.448*** (0.131)
Level of education				
Primary school and below	Reference			
Junior high school and above	−1.766*** (0.292)	−0.491*** (0.100)	−3.221*** (0.534)	−0.989*** (0.178)
Employment				
None	Reference			
Yes	0.122 (0.225)	0.119 (0.077)	−0.749* (0.404)	−0.180 (0.134)
Self-rated health				
Unhealthy	Reference			
Average	−1.540*** (0.251)	−0.412*** (0.086)	−1.876*** (0.438)	−0.489*** (0.145)
Healthy	−2.748*** (0.238)	−0.659*** (0.081)	−2.307*** (0.422)	−0.472*** (0.140)
Chronic disease				
None	Reference			
Yes	0.600** (0.251)	0.124 (0.085)	1.016** (0.424)	0.340** (0.141)
BADL	0.048 (0.073)	0.007 (0.025)	−0.026 (0.107)	0.001 (0.036)
Constant	4.612*** (1.441)	1.136** (0.491)	5.901** (2.312)	0.487 (0.768)
*R^2^*	0.221	0.192	0.233	0.212

*p < 0.1, **p < 0.05, ***p < 0.01.

## 4. Discussion

According to the results of the above empirical analysis, the higher the level of financial support provided by children, the more favorable it is for the older adult to maintain their mental health. Because rural China has long been characterized by a family care model for old age, with low levels of social security and support, the financial support of children has a greater impact on the quality of life of the older adult. In addition, the rural older adult have a stronger sense of raising children to provide against old age and increased emphasis on children giving back to their families, thus the more financial support they receive from their children, the more the older people’s sense of self-esteem and fulfillment is fulfilled, which reduces their negative emotions and makes it easier for them to keep their moods happy, the result that is in line with the previous study (33). The effect of children’s financial support on mental health was not as significant in the older adult without children’s migration as in the older adult with children’s migration, which may be since rural older adult without children’s migration receive lower levels of material support from their children, resulting in a weaker contribution of financial support to the health of the older adult.

The higher the level of life support from children, the higher the level of mental health of older persons. Older adult people’s physical fitness and self-care ability gradually decline due to physiological reasons, and they urgently need the help of their children, and their children’s care is more capable of satisfying their life needs compared to outsiders, thus the higher the level of psychological health of the older adult, and previous studies have also arrived at similar results (34). But in analyzing the heterogeneity of older adults according to the presence or absence of child migration, it was found that receiving life support from children effectively reduced the loneliness of older adults with migrated children, while it did not have a significant effect on the mental health of older adults without children’s migration. On the one hand, it may be because the level of children’s life support is higher for the older people without children’s migration, and children’s life care for the older people to some extent reduces the strength of the financial support provided by the children, which makes its effect on the mental health of the older people not obvious. On the other hand, in terms of emotional reasons, it may be because older people with children’s migration receive life support from their children, which means that the children may return home to visit their parents, and the meeting with their children makes the rural older people feel happy, and thus the life care has a more significant impact on the mental health of the older people.

The effect of children’s emotional support on the mental health of the older adult is not significant. This may be since older people living in rural areas have a restricted social circle and have a stronger need for emotional support from their children, but the “sense of loss” caused by the discrepancy between subjective needs and objective support may substantially reduce the role of emotional support in lowering the level of depression and diminishing the sense of loneliness.

Based on the results of the above analysis, it can also be seen that older people’s own characteristics have a greater impact on their levels of depression and loneliness than the impact of children’s support on their mental health, and that individual characteristics explain most of the variation in older people’s negative emotions.

The strength of this study lies in the large size of China’s rural population and the representativeness of research on the mental health of older adults. The limitations of this study mainly lie in the following: firstly, this paper only uses the simple method of multiple regression analysis to analyze the correlation between children’s support and mental health of rural older adult, and future research will try to use other methods to do a more comprehensive analysis. Secondly, although control variables were added where possible, there may still be omitted variables, and future research should test for potential confounders or mediators.

## 5. Conclusion

Based on data from the 2014 CLASS, this study used multiple linear regression to investigate the impact of intergenerational support from children on the mental health of older adults in rural China and analyzed the heterogeneity for older adults with different children’s migration status, and the following findings were derived from the empirical analysis: (1) The higher the level of financial support provided by children, the more favorable it is for rural older adults to maintain their mental health, which is more pronounced among the older adults with children’s migration. (2) The higher the level of children’s life support, the higher the mental health of rural older adult, but there is no significant effect of children’s life support on the mental health of the older adult without children’s migration. (3) Individual characteristics of older people have a greater impact on their mental health, the mental health of the rural older adult is higher among those who are male, have good marital status, high education level and better physical health.

According to the results of the above research, this paper draws the following results: Firstly, to better play the role of rural family care, rural grass-roots governments should actively publicize the culture of filial piety, forming a good atmosphere of love and respect for the older adult, and encouraging their children to provide support to meet the needs of the older adult. Secondly, the rural social security system should be improved, the level of rural old-age insurance should be raised appropriately, the level of medical services in rural areas should be raised, and village collectives should be used as the basis for introducing modes of onsite service and collective old-age care to satisfy the personalized needs for older adult care of the rural older adult, and to promote the sustainable development of both collective and family old-age care. Thirdly, social forces should actively utilize their advantages in resource allocation and combination to provide the rural older adult with cultural and recreational activities, legal counseling, mental health counseling, and other forms of services tailored to different needs, to ensure that the older adult have a sense of worthiness and enjoyment, and to continually improve the level of mental health of the older adult in rural areas.

## Data availability statement

Publicly available datasets were analyzed in this study. This data can be found at: http://class.ruc.edu.cn/index.php?r=index/index&hl=en.

## Author contributions

JL: conception and design, funding acquisition, and preparation. MJ and JL: methodology, writing—original draft and supervision. MJ, JL, ML, and AW: writing review and editing. All authors contributed to the article and approved the submitted version.

## Footnotes

^1^The main data results of the Seventh National Census. National Bureau of Statistics of China; (2021). Available online at: http://www.stats.gov.cn/sj/pcsj/rkpc/d7c/202303/P020230301403217959330.pdf (accessed on 11 May 2021).
