# Supplementary material for: The impact of adult children’s support on the psychological health of rural older adult people in China
Source: Front Public Health. 2023 Nov 6;11:1230580. doi: 10.3389/fpubh.2023.1230580 (PMC10657984; doi:10.3389/fpubh.2023.1230580)
Supplement: Supplementary file 1 [file Table_1.DOCX]

Supplementary Material

The Impact of Adult Children’s Support on the Psychological Health of Rural Elderly People in China

Juan Luo, Minglu Ji*, Mengyuan Li, Anning Wang

*** Correspondence:** Minglu Ji: jiminglu22@163.com

# Supplementary Tables

Table1 Cronbach's alpha for the Depression Scale and the Loneliness Scale

|  | Cronbach's alpha |
| --- | --- |
| Depression Scale | 0.917 |
| Loneliness Scale | 0.918 |

Table2 Variable definitions and assignments

| Variable settings | Assignment description |
| --- | --- |
| Dependent variable |  |
| Depression level | Never = 0; sometimes = 1; often = 2 (continuous variable, 0-18) |
| Loneliness | Never = 0; sometimes = 1; often = 2 (continuous variable, 0-18) |
| Independent variables |  |
| children's support |  |
| Economic support | Not given = 0; 1-199 yuan = 1; 200-499 yuan = 2; 500-999 yuan = 3 1000-1999 yuan = 4; 2000-3999 yuan = 5; 4000-6999 yuan = 6; 7000-11999 yuan = 7; 12000 yuan and above = 8(continuous variable, 0-40) |
| Life support | Almost nothing = 0; several times a year = 1; at least once a month = 2; at least once a week = 3; almost every day = 4(continuous variable, 0-20) |
| Emotional support | Often = 0; Sometimes = 1; Occasionally = 2; Never = 3(continuous variable, 0-15) |
| Control variables |  |
| Sociodemographic characteristics |  |
| Gender | Female = 0; male = 1 |
| Age | continuous variable, 57-98 years |
| Marital status | widowed/divorced/unmarried = 0; Married with spouse = 1 |
| Socioeconomic status |  |
| Educational attainment | primary school and below = 0; Junior high school and above = 1 |
| Employment | None = 0; Yes = 1 |
| Health status of the elderly |  |
| Self-rated health | Unhealthy = 0; General =1; Health = 2 |
| Chronic disease | None = 0; Yes = 1 |
| BADL | Can't move at all = 0; needs some help = 1; doesn't need help from others = 2 (continuous variable, 0-22) |

Table 3 Descriptive statistics of variables

| variable | collectivity | Have children migrated（N=3038） | Haven't children migrated（N=1047） |
| --- | --- | --- | --- |
| Dependent variables |  |  |  |
| Depression level (mean) | 9.80  （0.094） | 9.69  （0.108） | 10.13  （0.190） |
| Loneliness(mean) | 2.15  （0.031） | 2.11  （0.062） | 2.25  （0.036） |
| Independent variables |  |  |  |
| Children's support |  |  |  |
| Economic support (mean) | 12.08  （0.077） | 12.29  （0.090） | 11.44  （0.144） |
| Life support (mean) | 6.16  （0.055） | 5.69  （0.058） | 7.50  （0.120） |
| Emotional support (mean) | 6.96  （0.045） | 6.92  （0.052） | 7.09  （0.090） |
| Control variables |  |  |  |
| Sociodemographic characteristics |  |  |  |
| Gender（%） |  |  |  |
| 0 = female | 51.41% | 50.69% | 53.49% |
| 1 = male | 48.59% | 49.31% | 46.51% |
| Age(mean) | 70.39（0.125） | 70.23  （0.143） | 70.85  （0.257） |
| Marital status（%） |  |  |  |
| 0 = widowed/divorced/unmarried | 37.31% | 35.52% | 42.5% |
| 1 = Married with spouse | 62.69% | 64.48% | 57.5% |
| Socioeconomic status |  |  |  |
| Level of education（%） |  |  |  |
| 0 = primary school and below | 86.71% | 86.41% | 87.58% |
| 1 = Junior high school and above | 13.29% | 13.59% | 12.42% |
| Employment（%） |  |  |  |
| 0 = none | 67.05% | 66.06% | 69.91% |
| 1 = yes | 32.95% | 33.94% | 30.09% |
| Health status of the elderly |  |  |  |
| Self-rated health（%） |  |  |  |
| 0 = Unhealthy | 37.89% | 38.45% | 36.29% |
| 1 = Average | 25.04% | 24.82% | 25.69% |
| 2 = Healthy | 37.06% | 36.73% | 38.01% |
| Chronic disease（%） |  |  |  |
| 0 = none | 21.27% | 20.38% | 23.88% |
| 1 = yes | 78.73% | 79.62% | 76.12% |
| BADL (mean) | 11.16  （0.216） | 11.18  （0.238） | 11.11  （0.486） |

Table 4 Full-sample multiple linear regression results of children's support on the psychological welfare of rural elderly (N=4085)

| Variable | Depression level | Loneliness |
| --- | --- | --- |
| Economic support | -0.106*** | -0.038*** |
|  | (0.017) | (0.006) |
|  |  |  |
| Life support | -0.025 | -0.017** |
|  | (0.024) | (0.008) |
|  |  |  |
| Emotional support | 0.047 | 0.014 |
|  | (0.029) | (0.010) |
|  |  |  |
| Gender |  |  |
| Female | Reference |  |
| Male | -2.367*** | -0.679*** |
|  | (0.179) | (0.061) |
|  |  |  |
| Age | 0.122*** | 0.037*** |
|  | (0.012) | (0.004) |
|  |  |  |
| Marital status |  |  |
| Widowed/divorced/unmarried | Reference |  |
| Married with spouse | -0.990*** | -0.601*** |
|  | (0.198) | (0.067) |
|  |  |  |
| Level of education |  |  |
| Primary school and below | Reference |  |
| Junior high school and above | -2.104*** | -0.602*** |
|  | (0.256) | (0.087) |
|  |  |  |
| Employment |  |  |
| None | Reference |  |
| Yes | -0.101 | 0.044 |
|  | (0.196) | (0.066) |
|  |  |  |
| Self-rated health |  |  |
| Unhealthy | Reference |  |
|  |  |  |
| Average | -1.614*** | -0.427*** |
|  | (0.218) | (0.074) |
|  |  |  |
| Healthy | -2.632*** | -0.610*** |
|  | (0.207) | (0.070) |
|  |  |  |
| Chronic disease |  |  |
| None | Reference |  |
| Yes | 0.709*** | 0.188** |
|  | (0.215) | (0.073) |
|  |  |  |
| Self-care ability（ADL） | 0.021 | 0.005 |
|  | (0.060) | (0.020) |
|  |  |  |
| Constant | 4.969*** | 0.933** |
|  | (1.220) | (0.414) |
| R^2^ | 0.222 | 0.193 |

*p<0.1,**p<0.05,***p<0.01

Table 5 Grouped multiple linear regression results of children's support on the psychological welfare of rural elderly

| Variable | Have children migrated（N=3038） | | Haven't children migrated（N=1047） | |
| --- | --- | --- | --- | --- |
|  | Depression level | Loneliness | Depression level | Loneliness |
| Economic support | -0.117*** | -0.042*** | -0.069* | -0.026** |
|  | (0.020) | (0.007) | (0.037) | (0.012) |
|  |  |  |  |  |
| Life support | -0.049 | -0.032*** | -0.002 | 0.007 |
|  | (0.030) | (0.010) | (0.044) | (0.015) |
|  |  |  |  |  |
| Emotional support | 0.051 | 0.016 | 0.032 | 0.007 |
|  | (0.033) | (0.011) | (0.058) | (0.019) |
|  |  |  |  |  |
| Gender |  |  |  |  |
| Female | Reference |  |  |  |
| Male | -2.419*** | -0.670*** | -2.210*** | -0.695*** |
|  | (0.206) | (0.070) | (0.360) | (0.120) |
|  |  |  |  |  |
| Age | 0.126*** | 0.035*** | 0.112*** | 0.039*** |
|  | (0.014) | (0.005) | (0.024) | (0.008) |
|  |  |  |  |  |
| Marital status |  |  |  |  |
| Widowed/divorced/unmarried | Reference |  |  |  |
| Married with spouse | -0.942*** | -0.645*** | -1.030*** | -0.448*** |
|  | (0.229) | (0.078) | (0.395) | (0.131) |
|  |  |  |  |  |
| Level of education |  |  |  |  |
| Primary school and below | Reference |  |  |  |
| Junior high school and above | -1.766*** | -0.491*** | -3.221*** | -0.989*** |
|  | (0.292) | (0.100) | (0.534) | (0.178) |
|  |  |  |  |  |
| Employment |  |  |  |  |
| None | Reference |  |  |  |
| Yes | 0.122 | 0.119 | -0.749* | -0.180 |
|  | (0.225) | (0.077) | (0.404) | (0.134) |
|  |  |  |  |  |
| Self-rated health |  |  |  |  |
| Unhealthy | Reference |  |  |  |
|  |  |  |  |  |
| Average | -1.540*** | -0.412*** | -1.876*** | -0.489*** |
|  | (0.251) | (0.086) | (0.438) | (0.145) |
|  |  |  |  |  |
| Healthy | -2.748*** | -0.659*** | -2.307*** | -0.472*** |
|  | (0.238) | (0.081) | (0.422) | (0.140) |
|  |  |  |  |  |
| Chronic disease |  |  |  |  |
| None | Reference |  |  |  |
| Yes | 0.600** | 0.124 | 1.016** | 0.340** |
|  | (0.251) | (0.085) | (0.424) | (0.141) |
|  |  |  |  |  |
| BADL | 0.048 | 0.007 | -0.026 | 0.001 |
|  | (0.073) | (0.025) | (0.107) | (0.036) |
|  |  |  |  |  |
| Constant | 4.612*** | 1.136** | 5.901** | 0.487 |
|  | (1.441) | (0.491) | (2.312) | (0.768) |
| R^2^ | 0.221 | 0.192 | 0.233 | 0.212 |

*p<0.1, **p<0.05, ***p<0.01

# Stata code

cd"D:\桌面\小论文投稿\数据输出"

*清洗数据，筛选自己需要的变量并赋值

keep s05a a01 clea01 a02a clea02a a03 a04 b01 b0401 b0402 b0403 b0404 b0405 b0406 b0407 b0408 b0409 b0410 b0411 b11 c01 c12 e0201 e0202 e0203 e0204 e0205 e0206 e0207 e0208 e0209 e0210 e0211 e0212 f04a f04b f0401a f0401b f71001 f71002 f71003 f71004 f71005 f71301 f71601 f72201 f71302 f71602 f72202 f71303 f71603 f72203 f71304 f71604 f72204 f71305 f71605 f72205

gen resident=s05a

drop if missing(resident)

keep if s05a==5

gen age=2014-clea02a

drop if missing(age)

label variable age"age"

gen gender=clea01

drop if missing(gender)

label variable gender"gender"

replace gender=0 if gender==2

label define gender_value 0"女" 1 "男"

gen marriage=a03

drop if missing(marriage)

replace marriage=0 if marriage==2 | marriage==3 | marriage==4

label variable marriage"marriage"

label define marriage_value 0" widowed/divorced/unmarried" 1 "Married with spouse"

gen education=a04

drop if missing(education)

replace education=0 if education==1 | education==2 | education==3

replace education=1 if education==4 | education==5 | education==6

label variable education"education"

label define education_value 0 "primary school and below" 1"Junior high school and above"

gen self_rated_health=b01

drop if missing(self_rated_health)

replace self_rated_health=0 if self_rated_health==4 | self_rated_health==5

replace self_rated_health=2 if self_rated_health==1

replace self_rated_health=1 if self_rated_health==3

label variable self_rated_health"self_rated_health"

label define self_rated_health_value 0"unhealthy" 1"general" 2"healthy"

*将多分类变量生成虚拟变量

tab self_rated_health , gen(self_rated_health_1)

gen b0401_1=b0401

drop if missing(b0401_1)

replace b0401_1=0 if b0401_1==3

replace b0401_1=5 if b0401_1==1

replace b0401_1=2 if b0401_1==1

replace b0401_1=1 if b0401_1==5

gen b0402_1=b0402

drop if missing(b0402_1)

replace b0402_1=0 if b0402_1==3

replace b0402_1=5 if b0402_1==1

replace b0402_1=2 if b0402_1==1

replace b0402_1=1 if b0402_1==5

gen b0403_1=b0403

drop if missing(b0403_1)

replace b0403_1=0 if b0403_1==3

replace b0403_1=5 if b0403_1==1

replace b0403_1=2 if b0403_1==1

replace b0403_1=1 if b0403_1==5

gen b0404_1=b0404

drop if missing(b0404_1)

replace b0404_1=0 if b0404_1==3

replace b0404_1=5 if b0404_1==1

replace b0404_1=2 if b0404_1==1

replace b0404_1=1 if b0404_1==5

gen b0405_1=b0405

drop if missing(b0405_1)

replace b0405_1=0 if b0405_1==3

replace b0405_1=5 if b0405_1==1

replace b0405_1=2 if b0405_1==1

replace b0405_1=1 if b0405_1==5

gen b0406_1=b0406

drop if missing(b0406_1)

replace b0406_1=0 if b0406_1==3

replace b0406_1=5 if b0406_1==1

replace b0406_1=2 if b0406_1==1

replace b0406_1=1 if b0406_1==5

gen b0407_1=b0407

drop if missing(b0407_1)

replace b0407_1=0 if b0407_1==3

replace b0407_1=5 if b0407_1==1

replace b0407_1=2 if b0407_1==1

replace b0407_1=1 if b0407_1==5

gen b0408_1=b0408

drop if missing(b0408_1)

replace b0408_1=0 if b0408_1==3

replace b0408_1=5 if b0408_1==1

replace b0408_1=2 if b0408_1==1

replace b0408_1=1 if b0408_1==5

gen b0409_1=b0409

drop if missing(b0409_1)

replace b0409_1=0 if b0409_1==3

replace b0409_1=5 if b0409_1==1

replace b0409_1=2 if b0409_1==1

replace b0409_1=1 if b0409_1==5

gen b0410_1=b0410

drop if missing(b0410_1)

replace b0410_1=0 if b0410_1==3

replace b0410_1=5 if b0410_1==1

replace b0410_1=2 if b0410_1==1

replace b0410_1=1 if b0410_1==5

gen b0411_1=b0411

drop if missing(b0411_1)

replace b0411_1=0 if b0411_1==3

replace b0411_1=5 if b0411_1==1

replace b0411_1=2 if b0411_1==1

replace b0411_1=1 if b0411_1==5

gen BADL=b0401_1+b0402_1+b0403_1+b0404_1+b0405_1+b0406_1+b0407_1+b0408_1+b0409_1+b0410_1+b0411_1

label variable BADL"basic activities of daily living"

gen chronic_disease=b11

drop if missing(chronic_disease)

replace chronic_disease=0 if chronic_disease==2

label variable chronic_disease"chronic_disease"

label define chronic_disease_value 0 "none" 1"yes"

gen employment=c01

drop if missing(employment)

replace employment=0 if employment==2

label variable employment"employment"

label define employment_value 0 "none" 1"yes"

egen e0201_mean = mean(e0201)

replace e0201 = e0201_mean if e0201==9

replace e0201 = e0201_mean if missing(e0201)

egen e0203_mean = mean(e0203)

replace e0203 = e0203_mean if e0203==9

replace e0203 = e0203_mean if missing(e0203)

egen e0204_mean = mean(e0204)

replace e0204 = e0204_mean if e0204==9

replace e0204 = e0204_mean if missing(e0204)

egen e0205_mean = mean(e0205)

replace e0205 = e0205_mean if e0205==9

replace e0205 = e0205_mean if missing(e0205)

egen e0206_mean = mean(e0206)

replace e0206 = e0206_mean if e0206==9

replace e0206 = e0206_mean if missing(e0206)

egen e0207_mean = mean(e0207)

replace e0207 = e0207_mean if e0207==9

replace e0207 = e0207_mean if missing(e0207)

egen e0208_mean = mean(e0208)

replace e0208 = e0208_mean if e0208==9

replace e0208 = e0208_mean if missing(e0208)

egen e0209_mean = mean(e0209)

replace e0209 = e0209_mean if e0209==9

replace e0209 = e0209_mean if missing(e0209)

egen e0210_mean = mean(e0210)

replace e0210 = e0210_mean if e0210==9

replace e0210 = e0210_mean if missing(e0210)

replace e0201=0 if e0201==3

replace e0201=5 if e0201==1

replace e0201=2 if e0201==1

replace e0201=1 if e0201==5

replace e0204=0 if e0204==3

replace e0204=5 if e0204==1

replace e0204=2 if e0204==1

replace e0204=1 if e0204==5

replace e0209=0 if e0209==3

replace e0209=5 if e0209==1

replace e0209=2 if e0209==1

replace e0209=1 if e0209==5

replace e0203=0 if e0203==1

replace e0203=1 if e0203==2

replace e0203=2 if e0203==3

replace e0205=0 if e0205==1

replace e0205=1 if e0205==2

replace e0205=2 if e0205==3

replace e0206=0 if e0206==1

replace e0206=1 if e0206==2

replace e0206=2 if e0206==3

replace e0207=0 if e0207==1

replace e0207=1 if e0207==2

replace e0207=2 if e0207==3

replace e0208=0 if e0208==1

replace e0208=1 if e0208==2

replace e0208=2 if e0208==3

replace e0210=0 if e0210==1

replace e0210=1 if e0210==2

replace e0210=2 if e0210==3

gen depression_level=e0201+e0203+e0204+e0205+e0206+e0207+e0208+e0209+e0210

label variable depression_level"depression_level"

egen e0202_mean = mean(e0202)

replace e0202 = e0202_mean if e0202==9

replace e0202 = e0202_mean if missing(e0202)

egen e0211_mean = mean(e0211)

replace e0211 = e0211_mean if e0211==9

replace e0211 = e0211_mean if missing(e0211)

egen e0212_mean = mean(e0212)

replace e0212 = e0212_mean if e0212==9

replace e0212 = e0212_mean if missing(e0212)

replace e0202=0 if e0202==1

replace e0202=1 if e0202==2

replace e0202=2 if e0202==3

replace e0211=0 if e0211==1

replace e0211=1 if e0211==2

replace e0211=2 if e0211==3

replace e0212=0 if e0212==1

replace e0212=1 if e0212==2

replace e0212=2 if e0212==3

gen loneliness=e0202+e0211+e0212

label variable loneliness"loneliness"

drop if missing(f0401a)

drop if missing(f0401b)

gen children_number=f0401a+f0401b

drop if children_number==0

replace f71001=0 if missing(f71001)

replace f71001=0 if f71001==1 | f71001==2

replace f71001=1 if f71001==3 | f71001==4 | f71001==5 | f71001==6 | f71001==7

replace f71002=0 if missing(f71002)

replace f71002=0 if f71002==1 | f71002==2

replace f71002=1 if f71002==3 | f71002==4 | f71002==5 | f71002==6 | f71002==7

replace f71003=0 if missing(f71003)

replace f71003=0 if f71003==1 | f71003==2

replace f71003=1 if f71003==3 | f71003==4 | f71003==5 | f71003==6 | f71003==7

replace f71004=0 if missing(f71004)

replace f71004=0 if f71004==1 | f71004==2

replace f71004=1 if f71004==3 | f71004==4 | f71004==5 | f71004==6 | f71004==7

replace f71005=0 if missing(f71005)

replace f71005=0 if f71005==1 | f71005==2

replace f71005=1 if f71005==3 | f71005==4 | f71005==5 | f71005==6 | f71005==7

gen child_migration=f71001+f71002+f71003+f71004+f71005

replace child_migration=1 if child_migration>0

label variable child_migration"child_migration"

label define child_migration_value 0 "none" 1"yes"

gen f71301_1=f71301-1

egen f71301_1_mean = mean(f71301_1)

replace f71301_1 = f71301_1_mean if missing(f71301_1)

gen f71302_1=f71302-1

egen f71302_1_mean = mean(f71302_1)

replace f71302_1 = f71302_1_mean if missing(f71302_1)

gen f71303_1=f71303-1

egen f71303_1_mean = mean(f71303_1)

replace f71303_1 = f71303_1_mean if missing(f71303_1)

gen f71304_1=f71304-1

egen f71304_1_mean = mean(f71304_1)

replace f71304_1 = f71304_1_mean if missing(f71304_1)

gen f71305_1=f71305-1

egen f71305_1_mean = mean(f71305_1)

replace f71305_1 = f71305_1_mean if missing(f71305_1)

gen economic_support=f71301_1+f71302_1+f71303_1+f71304_1+f71305_1

label variable economic_support"economic_support"

gen f71601_1=f71601-1

replace f71601_1=6 if f71601_1==0

replace f71601_1=7 if f71601_1==1

replace f71601_1=1 if f71601_1==3

replace f71601_1=0 if f71601_1==4

replace f71601_1=4 if f71601_1==6

replace f71601_1=3 if f71601_1==7

egen f71601_1_mean = mean(f71601_1)

replace f71601_1 = f71601_1_mean if missing(f71601_1)

replace f71601_1 = f71601_1_mean if f71601_1==9

gen f71602_1=f71602-1

replace f71602_1=6 if f71602_1==0

replace f71602_1=7 if f71602_1==1

replace f71602_1=1 if f71602_1==3

replace f71602_1=0 if f71602_1==4

replace f71602_1=4 if f71602_1==6

replace f71602_1=3 if f71602_1==7

egen f71602_1_mean = mean(f71602_1)

replace f71602_1 = f71602_1_mean if missing(f71602_1)

replace f71602_1 = f71602_1_mean if f71602_1==9

gen f71603_1=f71603-1

replace f71603_1=6 if f71603_1==0

replace f71603_1=7 if f71603_1==1

replace f71603_1=1 if f71603_1==3

replace f71603_1=0 if f71603_1==4

replace f71603_1=4 if f71603_1==6

replace f71603_1=3 if f71603_1==7

egen f71603_1_mean = mean(f71603_1)

replace f71603_1 = f71603_1_mean if missing(f71603_1)

replace f71603_1 = f71603_1_mean if f71603_1==9

gen f71604_1=f71604-1

replace f71604_1=6 if f71604_1==0

replace f71604_1=7 if f71604_1==1

replace f71604_1=1 if f71604_1==3

replace f71604_1=0 if f71604_1==4

replace f71604_1=4 if f71604_1==6

replace f71604_1=3 if f71604_1==7

egen f71604_1_mean = mean(f71604_1)

replace f71604_1 = f71604_1_mean if missing(f71604_1)

replace f71604_1 = f71604_1_mean if f71604_1==9

gen f71605_1=f71605-1

replace f71605_1=6 if f71605_1==0

replace f71605_1=7 if f71605_1==1

replace f71605_1=1 if f71605_1==3

replace f71605_1=0 if f71605_1==4

replace f71605_1=4 if f71605_1==6

replace f71605_1=3 if f71605_1==7

egen f71605_1_mean = mean(f71605_1)

replace f71605_1 = f71605_1_mean if missing(f71605_1)

replace f71605_1 = f71605_1_mean if f71605_1==9

gen life_support=f71601_1+f71602_1+f71603_1+f71604_1+f71605_1

label variable life_support"life_support"

gen f72201_1=f72201-1

replace f72201_1=6 if f72201_1==0

replace f72201_1=5 if f72201_1==1

replace f72201_1=0 if f72201_1==4

replace f72201_1=1 if f72201_1==3

replace f72201_1=2 if f72201_1==5

replace f72201_1=3 if f72201_1==6

egen f72201_1_mean = mean(f72201_1)

replace f72201_1 = f72201_1_mean if f72201_1==9

replace f72201_1 = f72201_1_mean if missing(f72201_1)

gen f72202_1=f72202-1

replace f72202_1=6 if f72202_1==0

replace f72202_1=5 if f72202_1==1

replace f72202_1=0 if f72202_1==4

replace f72202_1=1 if f72202_1==3

replace f72202_1=2 if f72202_1==5

replace f72202_1=3 if f72202_1==6

egen f72202_1_mean = mean(f72202_1)

replace f72202_1 = f72202_1_mean if f72202_1==9

replace f72202_1 = f72202_1_mean if missing(f72202_1)

gen f72203_1=f72203-1

replace f72203_1=6 if f72203_1==1

replace f72203_1=5 if f72203_1==2

replace f72203_1=0 if f72203_1==4

replace f72203_1=1 if f72203_1==3

replace f72203_1=2 if f72203_1==5

replace f72203_1=3 if f72203_1==6

egen f72203_1_mean = mean(f72203_1)

replace f72203_1 = f72203_1_mean if f72203_1==9

replace f72203_1 = f72203_1_mean if missing(f72203_1)

gen f72204_1=f72204-1

replace f72204_1=6 if f72204_1==1

replace f72204_1=5 if f72204_1==2

replace f72204_1=0 if f72204_1==4

replace f72204_1=1 if f72204_1==3

replace f72204_1=2 if f72204_1==5

replace f72204_1=3 if f72204_1==6

egen f72204_1_mean = mean(f72204_1)

replace f72204_1 = f72204_1_mean if f72204_1==9

replace f72204_1 = f72204_1_mean if missing(f72204_1)

gen f72205_1=f72205-1

replace f72205_1=6 if f72205_1==1

replace f72205_1=5 if f72205_1==2

replace f72205_1=0 if f72205_1==4

replace f72205_1=1 if f72205_1==3

replace f72205_1=2 if f72205_1==5

replace f72205_1=3 if f72205_1==6

egen f72205_1_mean = mean(f72205_1)

replace f72205_1 = f72205_1_mean if f72205_1==9

replace f72205_1 = f72205_1_mean if missing(f72205_1)

gen emotional_support=f72201_1+f72202_1+f72203_1+f72204_1+f72205_1

label variable emotional_support"emotional_support"

*量表信度检验

alpha e0201 e0203 e0204 e0205 e0206 e0207 e0208 e0209 e0210 //depression_level

alpha e0202 e0211 e0212 //loneliness

*描述性统计

//基本特征

mean age

mean age if child_migration==0

mean age if child_migration==1

tab gender

tab gender if child_migration==0

tab gender if child_migration==1

tab marriage

tab marriage if child_migration==0

tab marriage if child_migration==1

tab education

tab education if child_migration==0

tab education if child_migration==1

tab self_rated_health

tab self_rated_health if child_migration==0

tab self_rated_health if child_migration==1

tab chronic_disease

tab chronic_disease if child_migration==0

tab chronic_disease if child_migration==1

tab employment

tab employment if child_migration==0

tab employment if child_migration==1

mean BADL

mean BADL if child_migration==0

mean BADL if child_migration==1

mean economic_support

mean economic_support if child_migration==0

mean economic_support if child_migration==1

mean life_support

mean life_support if child_migration==0

mean life_support if child_migration==1

mean emotional_support

mean emotional_support if child_migration==0

mean emotional_support if child_migration==1

mean depression_level

mean depression_level if child_migration==0

mean depression_level if child_migration==1

mean loneliness

mean loneliness if child_migration==0

mean loneliness if child_migration==1

//平均值和标准差

tabstat depression_level economic_support life_support emotional_support age gender resident education marriage health chronic_disease life_satisfication health_satisfication resident_pension commercial_pension, s(mean sd) f(%12.3f) c(s)

*最小二乘法OLS回归输出

reg depression_level economic_support life_support emotional_support age gender marriage education employment self_rated_health_12 self_rated_health_13 chronic_disease BADL

estimates store m1

esttab m1 using qvanyiyu.rtf, replace star (* 0.1 ** 0.05 *** 0.01) b(%6.3f) r2 se

*方差膨胀因子检验

estat vif

reg loneliness economic_support life_support emotional_support age gender marriage education employment self_rated_health_12 self_rated_health_13 chronic_disease BADL

estimates store m2

esttab m2 using qvangudu.rtf, replace star (* 0.1 ** 0.05 *** 0.01) b(%6.3f) r2 se

*方差膨胀因子检验

estat vif

*异质性分析

//子女迁移

reg depression_level economic_support life_support emotional_support age gender marriage education employment self_rated_health_12 self_rated_health_13 chronic_disease BADL if child_migration==1

estimates store m3

esttab m3 using yesyiyu.rtf, replace star (* 0.1 ** 0.05 *** 0.01) b(%6.3f) r2 se

reg loneliness economic_support life_support emotional_support age gender marriage education employment self_rated_health_12 self_rated_health_13 chronic_disease BADL if child_migration==1

estimates store m4

esttab m4 using yesgudu.rtf, replace star (* 0.1 ** 0.05 *** 0.01) b(%6.3f) r2 se

reg depression_level economic_support life_support emotional_support age gender marriage education employment self_rated_health_12 self_rated_health_13 chronic_disease BADL if child_migration==0

estimates store m5

esttab m5 using noneyiyu.rtf, replace star (* 0.1 ** 0.05 *** 0.01) b(%6.3f) r2 se

reg loneliness economic_support life_support emotional_support age gender marriage education employment self_rated_health_12 self_rated_health_13 chronic_disease BADL if child_migration==0

estimates store m6

esttab m6 using nonegudu.rtf, replace star (* 0.1 ** 0.05 *** 0.01) b(%6.3f) r2 se
